# Mini Hyrax vs Hyrax expanders in the rapid palatal expansion in adolescents with posterior crossbite: a randomized controlled clinical trial

**DOI:** 10.1186/s40510-021-00365-5

**Published:** 2021-09-01

**Authors:** Giordani Santos Silveira, Lucas Guimarães Abreu, Juan Martin Palomo, Larissa Salgado da Matta Cid Pinto, Adriana Alkmim de Sousa, Bruno Frazão Gribel, Dauro Douglas Oliveira

**Affiliations:** 1 School of Dentistry, Pontifical Catholic University of Minas Gerais, Belo Horizonte, MG Brazil; 2 Department of Pediatric Dentistry, Federal University of Minas Gerais, Belo Horizonte, MG Brazil; 3 Department of Orthodontics, Case Western Reserve University, Cleveland, OH USA; 4 Private Practice, Belo Horizonte, MG Brazil

**Keywords:** Posterior crossbite, Adolescent, Palatal expansion technique, Quality of life, Randomized clinical trial

## Abstract

**Purpose:**

The aim of this study was to compare the dental effects, impact on quality of life, and pain perception of adolescents wearing Mini Hyrax and Hyrax expanders in rapid palatal expansion.

**Methods:**

Thirty-four adolescents aged 11 to 16 years, with maxillary transverse deficiency (unilateral or bilateral posterior crossbite), were randomly allocated into two groups, Mini Hyrax group and Hyrax group (1:1 ratio). Dental effects were evaluated by digitally superimposed pretreatment and postretention three-dimensional intraoral scans on the palatal rugaes using the software 3DSlicer. Impact on quality of life was assessed with the OHIP-14 questionnaire applied in the pretreatment, posttreatment and postretention. Visual analog scale was applied 24, 48, and 72 h and 7 days after the first activation of the expander.

**Results:**

Thirty of the 34 adolescents recruited completed the study. There were no statistically significant differences in dentoalveolar effects between groups. OHIP-14 scores across time among Mini Hyrax wearers were similar to those of the Hyrax wearers. The inter-group comparisons showed no difference between groups with respect to the OHIP-14 scores in posttreatment and postretention (*p* > 0.05). There were no differences in pain perception between groups. Considering intra-group comparison, the reduction in pain perception among adolescents in the Mini Hyrax group was gradual. Among adolescents in the Hyrax group, a statistically significant reduction between 48 and 72 h was observed.

**Conclusion:**

There were no significant differences in dental effects, impact on quality of life and pain perception between adolescents wearing Mini Hyrax and Hyrax expanders in rapid palatal expansion.

## Introduction

Maxillary constriction is the result of deficiency in the growth of the maxilla in the transverse direction. The most representative clinical finding of maxillary constriction is a unilateral or bilateral posterior crossbite [1]. Among adolescents, the prevalence of this condition ranges between 10% and 15% [2], with no differences regarding individuals’ sex or ethnicity [3]. Orthopedic maxillary expansion by means of the opening of the midpalatal suture is the routine procedure for the correction of maxillary constriction in growing patients [4, 5]. Haas-type and Hyrax are the most widely used expansion devices for this purpose [4]. Both expanders produce similar dentoskeletal effects [4, 6], but therapy with Hyrax causes less irritation on the palate because it has no acrylic pad that injuries the mucosa of the palatal vault during expansion making oral hygiene easier [7]. Patients undergoing rapid palatal expansion (RPE) with these bonded expanders may experience limitations in chewing, swallowing and speech [8–11].

In order to provide greater comfort and less impairment of speech and oral hygiene practices, a 2-point palatal expander using Hyrax jackscrew with two arms (two mesial arms cut-off) and anchorage only in the first permanent molars was introduced as an alternative to Hyrax for the treatment of individuals in mixed dentition and in the early phase of permanent dentition [12]. Though efficacious in promoting the expansion of the upper arch and alveolar process as well as the opening of the medial palatine suture [12, 13], this expander showed less stable results at the initial phase of the expansion treatment when compared with Hyrax [12].

A comparative study introduced another tooth-borne expander; the two-arm Hyrax, with improvement of the dental anchorage including anterior extension of the arms bilaterally and contour of the palatal surfaces of the premolars [14]. It has been demonstrated that the two-arm Hyrax provokes less speech impairment than the four-arm Hyrax during RPE [14]. Orthodontic suppliers have provided a reduced volume jackscrew with two arms which allows one to manufacture the 2-arm tooth-borne expander with extended anchorage on the premolars that could be recognized as the Mini Hyrax.

To date, no comparison of the dental effects of treatment with Mini Hyrax and treatment with Hyrax and/or Haas expanders has been found in the literature to answer the question: does the absence of 2 anterior arms in Mini Hyrax provoke different expansion in maxillary arch than that obtained with 4 arms expanders? In addition, the assessment of the impact on patients’ quality of life and the perception of pain during the wearing of the expander devices have not been in the scope of dental research as well. Therefore, the aim of this study was to compare the dental effects of Mini Hyrax with those of Hyrax after RPE in adolescents in a randomized controlled clinical trial that also assessed the impact of treatment on individuals’ quality of life and their perception of pain. The null hypothesis was that there were no differences regarding dental effects, impact on quality of life, and pain perception between wearers of Mini Hyrax and wearers of Hyrax.

## Subjects and methods

### Trial design and changes after trial commencement

This randomized controlled clinical trial with 2-parallel arms followed the guidelines of Consolidated Standards of Reporting Trials-2010 [15]. There was a change in the methods after trial commencement to improve the way how angular changes of teeth were measured, according to the software used in this study.

### Participants, eligibility criteria, and settings

This study was registered in the ClinicalTrials.gov (NCT03846518) in December 2018, after recruitment of participants was concluded. Approval of the Research Ethics Committee of the Pontíficia Universidade Católica de Minas Gerais was obtained under protocol number: 84651618.0.0000.51370.

The recruitment of participants was conducted at Department of Dentistry of the Pontifícia Universidade Católica de Minas Gerais and at public schools close to the university. The inclusion criteria were individuals in permanent dentition with transverse maxillary deficiency and uni- or bilateral crossbite. The correct diagnosis of the transverse maxillary deficiency excluded cases with only dental posterior crossbite (vestibular inclination of the crowns of the mandibular posterior teeth and/or palatal inclination of the crowns of the posterior maxillary teeth). Restrictions on ethnicity or sagittal and vertical malocclusion were not imposed in anyway. The following exclusion criteria were applied: individuals who were 17 years or older, individuals with missing teeth (except canines and upper second molars), dental caries, periodontal disease, cleft lip and palate, syndromes, and those reporting previous orthodontic treatment.

### Interventions

**Fig. 1:**
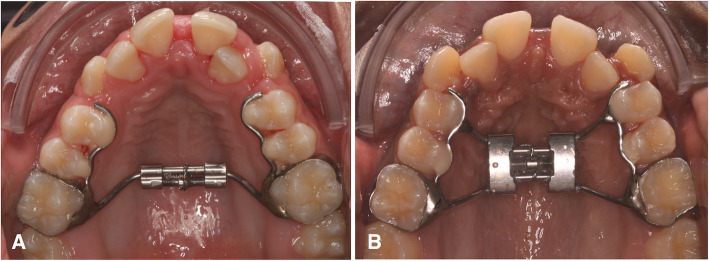
Expander appliances: **A**. Mini Hyrax; **B**. Hyrax

In the experimental group, the mini expander jackscrew (Dynaflex, Saint Ann, USA) was used and in the comparison group, the Hyrax jackscrew (Morelli, Sorocaba, Brazil) was used. The expanders had bands placed on the first molars. The expansion jackscrew size of the Mini Hyrax was 8 mm and of the Hyrax was 9 mm. The positioning of the expander jackscrew was standardized: in the occlusal view, perpendicular to the median palatal raphe, between the second premolars and the first molars; and in the vertical view, parallel to the occlusal plane, 3 mm apically to the palatal gingival contour of the first permanent molars. After placement of the device, the first and second premolars were bonded to the expanders with composite resin (Fig. 1). The activation protocol was identical for both expanders: 2 turns per day until the overcorrection of the crossbite was obtained. Overcorrection was characterized by the contact of the tips of the palatal cusps of the upper molars with the tips of the buccal cusps of the lower molars. Then, the device was maintained in position for 6 months.

### Outcomes

In this study, the main outcome was the correction of the transverse discrepancy, clinically characterized by the posterior crossbite.

#### Dental effects (primary and secondary outcomes)

Intraoral scans were performed with the intraoral scanner (TRIOS, 3Shape, Copenhagen, Denmark) in the pre-treatment (T0) and posttreatment time, after the 6-month retention period had been completed, until 4 h after the removal of the expander (T2). Digital models in .stl file format were obtained. The primary outcome was transverse linear measurement of the first molars. The secondary outcomes were transverse linear measurement of the first and second premolars; rotation of the first and second premolars, and first molars; buccolingual inclination of the first and second premolars, and first molars.

#### Impact on quality of life (secondary outcome)

The short version of the Oral Health Impact Profile (OHIP-14) questionnaire validated in the Brazilian portuguese [16] was applied to participants in the pre-treatment (T0), on the 14th day of the expander activation (T1), and posttreatment (T2) when the 6-month retention period had been completed. The OHIP-14 has 14 questions distributed across seven domains: functional limitation, physical discomfort, psychological discomfort, physical disability, psychological disability, social disability, and handicap. The scores for each domain and the total score were evaluated. A higher score indicates a more negative perception of the individual with respect his/her quality of life.

#### Pain perception (secondary outcome)

Patients were asked about the highest pain level they had experienced in 24 h, 48 h, 72 h, and 7 days, during the period of activation of the expanders. The instrument used for pain measurement was the visual analog scale (VAS) of 100 mm in length printed on paper in four units. Each participant was instructed to mark with a pen the point that represented the intensity of pain felt in each of the four times, with the extreme left end representing “no pain” and the extreme right end representing “worst possible pain” [17]. The measurement of the distance from the left end of the horizontal line to the marking made by the participants was performed with a digital caliper (Mitutoyo 500-196-30B, Kawasaki, Japan).

### Digital model measurement method

#### Model orientation

The pre-treatment digital models in the .stl file format were oriented in the three planes of the space using the “Transforms” tool of the 3D Slicer open source software (version 4.8.0, available at www.slicer.org). Initially, these models were positioned so that the mesiopalatal cusps of the first molars, bilaterally, and the midpoint of the incisal border of the upper right central incisor touched the axial plane. In an occlusal view, the median palatal raphe was positioned coincidentally to the sagittal plane with the incisive papilla coinciding with the coronal plane.

#### Model approximation

The posttreatment models were approximated to the oriented pre-treatment models also by the “Transforms” tool. This approximation was necessary so that the software’s algorithm could perform the three-dimensional superimposition of the models.

#### Model superimposition

Seven landmarks on the palate were marked using the “Surface Registration/Add and Move Landmarks” tool of the 3D Slicer software. The selected points were: medial edges of the second rugae, medial edges of the third rugae, midpoint on the third rugae, located in the median palatal raphe; and two points also located on the median palatal raphe, 5 mm and 10 mm posterior to the midpoint of the third rugae.

**Fig. 2:**
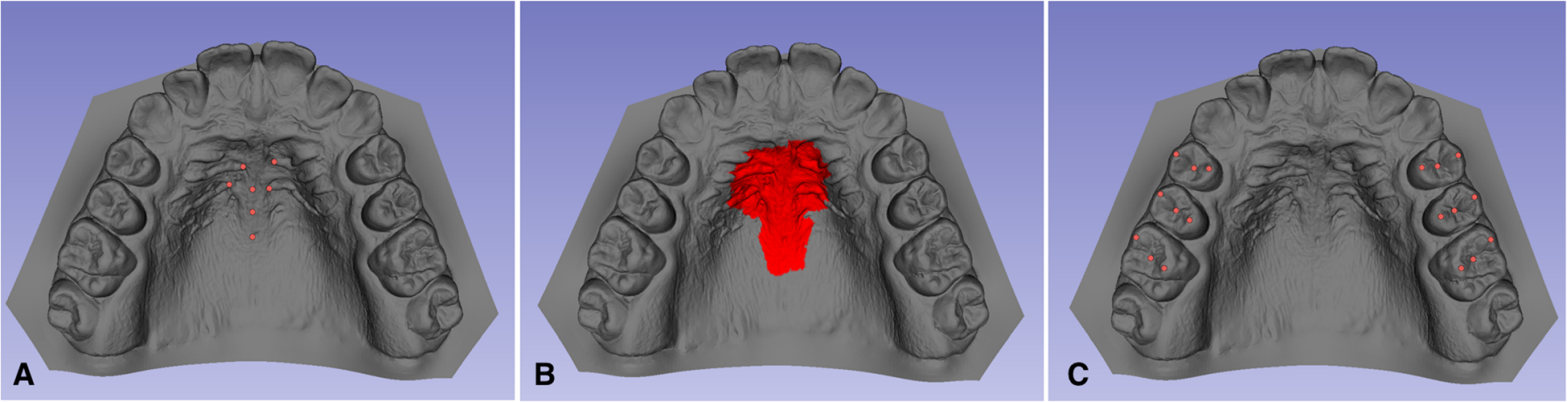
**A** Seven landmarks were used for landmark registration on the palatal rugae. **B** Region of interest around the seven landmarks used for ROI registration. **C** Landmarks used for dental measurements

**Fig. 3:**
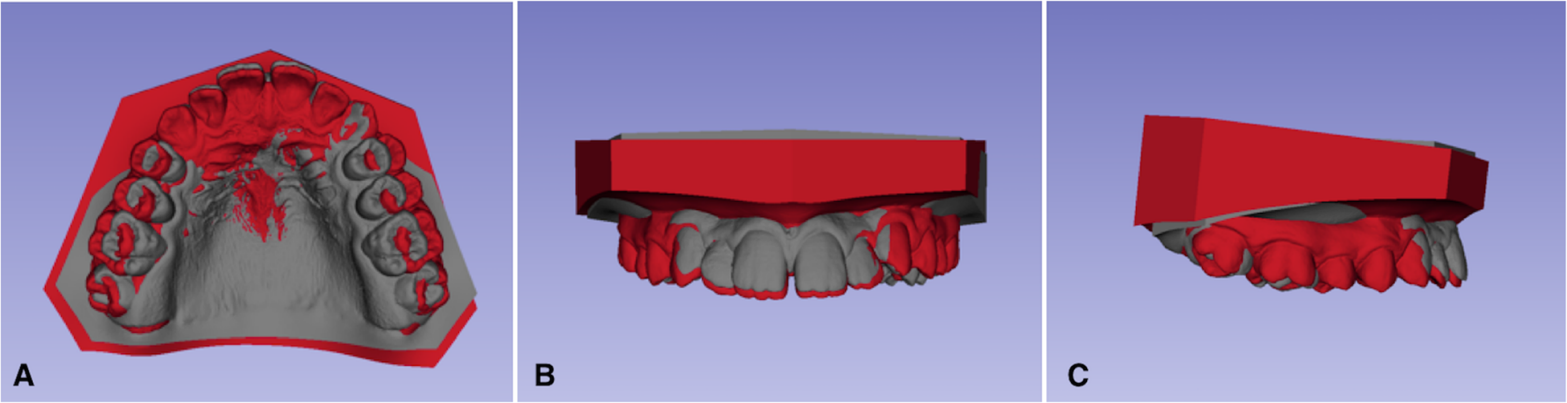
Superimposition of the pre-treatment (gray) and posttreatment (red) digital models. **A** Anterior view. **B** Frontal view. **C** Lateral view

When a point is selected, the vertex of one of thousands of triangles that form the surface mesh of the digital model is marked. As the marking of the same vertex on two models in two stages is unlikely [18], the superimposition was carried out using the region of interest (ROI). For each of the seven points above, an ROI size of 15 was given, that is, the program included 15 layers of triangles around the vertex of the triangle selected by the researcher. The superimposition of the T0 and T2 models was based on the total surface area resulting from the sum of the ROIs of each of the points (Figs. 2 and 3).

#### Three-dimensional quantitative measurements

Using the tool “Q3DC/Add and Move Landmarks” of the 3D Slicer software, the following reference points were marked in the pre-treatment and posttreatment digital models: tip of the buccal and palatal cusps of the first and second premolars, tip of the mesial cusps-vestibular and mesio-palatal of the first molars, centroid (midpoint of the meso-distal groove of the occlusal surface) of the first and second premolars and centroid (point of intersection of the vestibulo-occlusal groove with the mesio-distal groove of the occlusal surface) of the first molars (Fig. 2).

From these points, linear and angular measurements were performed with the 3D Slicer software using the tools “Q3DC/Calculate Distance Between Two Landmarks” and “Q3DC/Calculate Angle Between Two Lines,” respectively. Angles formed by the clockwise displacement of lines in the considered plane (axial and coronal) had positive values; if counterclockwise, negative values were provided.

For the transverse linear measurements of the first and second premolars (secondary outcomes), and of the first molars (primary outcome), the distance between the centroid points for each pair of homologous teeth, both at T0 and T2 was considered. The angular measurements performed by the 3D Slicer software can be decomposed in the three planes of the space to determine the “Pitch,” “Roll,” and “Yaw.” In view of the objectives of this research, the following angular measures (secondary outcomes) were recorded:

a) Rotation of the first and second premolars: angle formed in the axial plane between the lines resulting from the connection of the tips of the buccal and palatal cusps at T0 and T2 (“Yaw”).

b) Rotation of the first molars: angle formed in the axial plane between the lines resulting from the connection of the tips of the mesiobuccal and mesiopalatal cusps in T0 and T2 (“Yaw”).

c) Buccolingual inclination of the first and second premolars: angle formed in the coronal plane between the lines resulting from the connection of the tips of the buccal and palatal cusps in T0 and T2 (“Roll”).

d) Buccolingual inclination of the first molars: angle formed in the coronal plane between the lines resulting from the connection of the tips of the mesio-buccal and mesiopalatal cusps in T0 and T2 (“Roll”).

### Sample size calculation

The sample size was calculated prospectively prior to trial onset. Considering the standard deviation of 0.9 mm [4], a test power of 80% and a significance level of 5% to demonstrate a difference of 1.0 mm in the inter-molar distance between individuals treated with Mini Hyrax and those treated with Hyrax, the minimum sample size was determined in 28 participants; 14 individuals in each group. To compensate for possible losses, three individuals were added to each group.

### Randomization

Thirty-four individuals (17 adolescents in each group, 8 males and 9 females) participated. Before the beginning of the screening of the participants, a stratified randomization by sex was performed electronically generated by the program “Random Allocation Software” [19]. The individuals were randomly distributed (ratio 1: 1) into the two groups. Allocation concealment of the participants was guaranteed through the sequential opening of opaque envelopes containing the name of the expander that each participant would wear. These envelopes were previously sealed and sequentially numbered for each sex by a staff member who was unaware of the study’s method.

### Blinding

Blinding of the researchers who performed the treatments was unfeasible, but blinding of the researcher who had performed the measurements was guaranteed. The researcher did not have access to any information that allowed him to identify to which intervention group the digital models, quality of life questionnaires, and visual analog scales belonged. The participants were not informed about what type of expander they were wearing.

### Statistical analyses

Descriptive statistics of participants’ age (mean and standard deviation), sex, posterior crossbite, and angle classification was conducted. The Kolmogorov-Smirnov test was employed to assess the distribution of quantitative data on the dental effects OHIP-14 scores and VAS. For the dental effects and the OHIP-14 scores, the results of the normality test were *p* > 0.05. Data presented normal distribution and parametric tests were used. For VAS, the results of the normality test were *p* < 0.05. Data presented non-normal distribution and non-parametric tests were used. The Pearson’s chi-square test and Fisher’s exact test were used for comparisons between groups with respect to adolescents’ sex, posterior crossbite, and angle classification. The Student *t* test (independent samples) was used for comparison between groups with respect to adolescents’ age and number of activations of the orthodontic device. The Student *t* test (independent samples) was employed to assess whether any difference between groups regarding the dental outcomes and OHIP-14 scores at baseline (T0) existed. In addition, the chi-square test and the Student *t* test were deployed to compare excluded individuals and individuals who participated in the entire trial regarding sex, age, posterior crossbite, and angle classification as well as transverse linear measurements and quality of life at T0. The level of significance was set at *p* < 0.05.

The calculation of the error of the method was performed for the measurements of transverse distances, rotation, inclination, and vertical displacement. These measures were evaluated in nine individuals twice with an interval of 30 days between the first and the second evaluation. The calculation of the systematic error of the method was done by means of the intra-class correlation coefficient (ICC). To calculate the random error, the Dahlberg formula was used [20].

To calculate the differences between the Mini Hyrax and Hyrax groups with respect to the linear and angular measurements, the Student *t* test (independent samples) was used. Regression analysis assessing differences between Mini-Hyrax and Hyrax groups with respect to dental changes (T2–T0), controlling for the dental measures at T0, participants’ age, and number of activations of the appliance was also performed. Effect size and 95% confidence intervals (CI) were provided. The effect size was also calculated dividing the mean difference between the Mini Hyrax and Hyrax groups by the pooled standard deviation. A value of 0.20 represented a small effect size, 0.50 denoted a medium effect size and 0.80 indicated a large effect size [21].

For intra-group comparisons of data of OHIP-14 at different times (T0, T1 and T2), the ANOVA test for repeated measures was applied. The paired *t* test was used for comparisons of pairs (T0 X T1, T0 X T2, T1 X T2) in both groups. Bonferroni correction was applied. The level of significance was *p* < 0.016. Comparisons of OHIP-14 scores at T1 and OHIP-14 scores at T2 between Mini Hyrax and Hyrax groups, controlling for the OHIP-14 scores at T0 were performed with a regression analysis. In this final analysis, coefficients and 95% CI were provided. The significance was set at *p* < 0.05.

For the results of VAS, the Wilcoxon test was applied in the intra-group comparisons of times. In the inter-group comparison between expander wearers, the Mann-Whitney test was used instead. In the inter-group comparison, the effect size was also calculated dividing the mean difference between the Mini Hyrax and Hyrax groups by the pooled standard deviation. A value of 0.20 represented a small effect size, 0.50 denoted a medium effect size and 0.80 indicated a large effect size. Effect size was provided along with 95% CI. The level of significance was set at *p* < 0.05. All tests were run in SPSS program, version 20.0 (SPSS Inc., Chicago, USA).

## Results

**Fig. 4:**
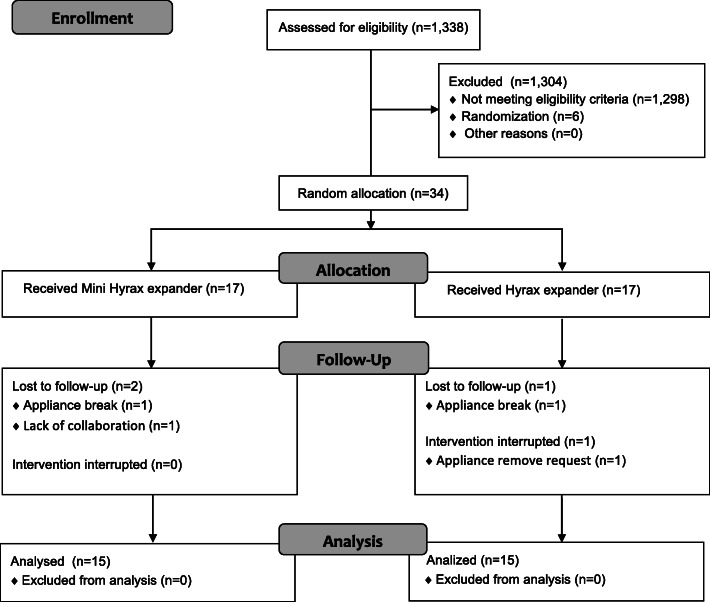
CONSORT diagram showing patient flow during the trial

A total of 1338 individuals from the Department of Dentistry and 6 public schools in the same region underwent intraoral clinical examination from April to November 2018. Forty individuals met the inclusion criteria. Thirty-four individuals, 16 boys and 18 girls, participated. The age of individuals ranged from 11 to 16 years. Of the 34 individuals, 4 were excluded (Fig. 4). No difference between excluded individuals and individuals who participated in the trial for sex (*p* = 0.348), age (*p* = 0.06), posterior crossbite (*p* = 0.99), angle classification (*p* = 0.765), transverse linear molar measurements at T0 (*p* = 0.384), and the total score of OHIP-14 at T0 (*p* = 0.375) was observed. The treatments were performed from February 2019 to February 2020, and outcome measurements were performed from March 2019 to February 2020, when the retention period of the last participant had been finished.

Considering the variables sex, age, type of posterior crossbite, angle classification, and dental outcomes, there were no statistically significant differences between groups in baseline. No difference between groups regarding number of activations of the orthodontic device was observed as well (*p* > 0.05). Distribution of sex, type of posterior crossbite, and angle classification is showed in Table 1. Mean age of participants in the Mini Hyrax group was 13.50 (± 1.39) years and in the Hyrax group was 13.83 (± 1.26) years (*p* = 0.51). The mean number of activations among Mini Hyrax wearers was 30.67 (± 1.49) and among Hyrax wearers was 31.27 (± 2.86) (*p* = 0.48). In the Mini Hyrax group, after the losses, eight males and seven females remained in the study, and in the Hyrax group, seven males and eight females remained (*p* = 0.99). The values of transverse distances in the Mini Hyrax and Hyrax group were, respectively, inter first premolars 34.35 (± 2.46) mm and 33.85 (± 2.0) mm (*p* = 0.58), inter seconds premolars of 38.62 (± 5.33) mm and 39.76 (± 3.34) mm (*p* = 0.48), and inter-first molars of 46.12 (± 3.27) and 45.76 (± 3.96) mm (*p* = 0.79).

**Table 1:** Distribution of adolescents regarding sex, posterior crossbite, and angle classification

	Mini Hyrax	Hyrax	*p* value
	N (%)	N (%)	
Sex			
Male	8 (53.3)	7 (46.7)	1.000^a^
Female	7 (46.7)	8 (53.3)	
Posterior crossbite			
Bilateral	5 (33.3)	4 (26.7)	0.811^b^
Unilateral right side	6 (40.0)	5 (33.3)	
Unilateral left side	4 (26.7)	6 (40.0)	
Angle classification			
Class I	5 (33.3)	6 (40.0)	1.000^b^
Class II	4 (26.7)	4 (26.7)	
Class III	6 (40.0)	5 (33.3)	

^a^Pearson’s chi-square test

^b^Fisher’s exact test

The results of the ICC for the measurements of transverse distances, rotation and inclination showed values above 0.90 indicating the absence of systematic error of the method. Only one measurement was 0.80. The calculation showed a random error between 0.01 mm and 0.23 mm for transverse distances, between 0.02^o^ and 3.1^o^ for rotation measurements, between 0.23^o^ and 1.77^o^ for measurements inclination.

The opening of the diastema between the maxillary central incisors was observed in all 30 individuals during the activation phase of the expanders, which represents the clinical sign of split of the midpalatal suture. Both expanders promoted a significant increase in transverse distances between the first premolars, between the second premolars and between the first upper molars (Table 2). The angular differences in rotation and buccal-lingual inclination were performed between pre-treatment and posttreatment lines (based in reference points) in the 3D Slicer program. Therefore, there were no values recorded separately at T0 and T2.

**Table 2:** Comparison of the changes during treatment (T2–T0) between the two groups

Measurements	Differences between groups T0 Mean (SD)^**a**^	Mini Hyrax-group T2–T0 Mean (SD)	Hyrax-group T2–T0 Mean (SD)	Difference between groups Mean (SD)	***p*** value^**a**^	Effect size	CI (95%)	Coefficient (95% CI)/***p*** value^**b**^
Distance 14–24 (mm)	0.50 (3.55)^c^	6.55 (0.72)	6.51 (1.19)	0.04 (0.90)	0.914	0.04	− 0.66–0.74	− 0.17 (− 0.87–0.52)/0.608
Distance 15–25 (mm)	− 1.14 (7.19)^c^	6.38 (0.64)	6.09 (1.01)	0.29 (0,86)	0.347	0.34	− 0.36–1.04	− 0.33 (− 0.94–0.28)/0.276
Distance 16–26 (mm)	0.35 (5.51)^c^	6.23 (0.68)	5.93 (0.75)	0.30 (0.72)	0.272	0.41	− 0.29–1.11	− 0.27 (− 0.78–0.24)/0.290
Rotation 14 (^o^)	− 3.09 (12.92)^c^	0.76 (5.48)	0.27 (5.30)	0.49 (5.34)	0.803	0.09	− 0.61–0.79	− 1.06 (− 5.08–2.94)/0.588
Rotation 24 (^o^)	− 1.18 (11.09)^c^	0.62 (5,98)	− 0.84 (6.11)	1.46 (5.92)	0.512	0.24	− 0.46–0.94	− 1.14 (− 5.90–3.60)/0.623
Rotation 15 (^o^)	− 6.31 (13.23)^c^	1.05 (6.06)	1.77 (3.03)	− 0.72 (4.12)	0.682	0.15	− 0.55–0.85	0.83 (− 3.09–4.76)/0.666
Rotation 25 (^o^)	− 1.85 (13.36)^c^	− 1.05 (5.77)	0.48 (3.31)	− 1.53 (4.22)	0.380	0.32	− 0.38–1.02	2.05 (− 1.08–5.19)/0.190
Rotation 16 (^o^)	− 3.29 (10.02)^c^	0.07 (2.57)	− 1.10 (3.94)	1.17 (3.36)	0.341	0.35	− 0.35–1.05	− 1.06 (− 3.78–1.65)/0.427
Rotation 26 (^o^)	− 4.29 (10.09)^c^	0.67 (3.55)	− 0.68 (3.83)	1.35 (3.61)	0.322	0.36	− 0.34–1.06	− 2.26 (− 4.68–0.16)/0.067
Inclination 14 (^o^)	0.39 (7.49)^c^	10.81 (5.84)	11.27 (5.25)	-0.46 (5.12)	0.822	0.08	− 0.62–0.78	0.14 (− 4.01–4.30)/0.943
Inclination 24 (^o^)	0.59 (8.16)^c^	11.78 (5.09)	9.25 (6.31)	2.53 (5.89)	0.238	0.43	− 0.27–1.13	− 2.76 (− 7.07–1.54)/0.199
Inclination 15 (^o^)	0.34 (7.29)^c^	10.16 (4.19)	10.43 (3.41)	-0.27 (3.29)	0.848	0.07	− 0.63–0.77	0.02 (− 2.92–2.96)/0.989
Inclination 25 (^o^)	− 0.18 (12.66)^c^	12.17 (3.35)	9.47 (3.75)	**2.70 (3.46)**	**0.047**	0.71	0.01–1.41	− 2.68 (− 5.42to − 0.04)/0.049
Inclination 16 (^o^)	− 2.09 (10.41)^c^	1.75 (4.45)	1.53 (3.03)	0.22 (3.98)	0.876	0.05	− 0.65–0.75	− 0.52 (− 3.47–2.41)/0.715
Inclination 26 (^o^)	0.60 (13.90)^c^	2.79 (3.51)	1.79 (2.90)	1.00 (3.26)	0.404	0.31	− 0.39–1.01	− 0.84 (− 3.33–1.63)/0.489

^a^Student *t* test (independent samples). Level of significance *p* < 0.05. *SD* standard deviation, *CI* confidence intervals, *disp* displacement, *mm* milimeters, ^o^ degrees. Rotation measurements: means with negative sign indicates counterclockwise rotation, Inclination measurements: mean with negative sign indicates lingual inclination of teeth on the right side and buccal inclination of teeth on the left side

^b^Regression analysis assessing differences between Mini-Hyrax and Hyrax groups of the dental changes (T2–T0), controlling for the dental measures at T0, participants’ age and number of activations of the appliance. Level of significance *p* < 0.05

^c^Denotes no difference between groups. *p* > 0.05

When the dental effects of the two expanders were compared, it was impossible to observe differences between groups, except for the buccolingual inclination of tooth 25 (*p* = 0.047). The effect of the expanders on tooth rotation was low (maximum mean difference was 1.77^o^) and did not present a specific pattern, given the size of the standard deviations. The first and second premolars showed an increase in the buccal inclination approximately in 10^o^, while the molars had values close to 2^o^. In the regression analysis assessing differences between Mini-Hyrax and Hyrax groups of the dental changes (T2–T0), controlling for the dental measurements at T0, participants’ age, and number of activations of the appliance, the results remained (Table 2).

The comparison between Mini Hyrax and Hyrax groups with respect to the OHIP-14 scores at T0 showed a significant difference only in the psychological disability domain (*p* = 0.012). For the other domains and the OHIP-14 total score, no difference was observed (*p* > 0.05) (Table 3). The results of the intra-group comparisons for the seven domains and for the total OHIP-14 score are displayed in Table 4. The intra-group comparisons revealed that the OHIP-14 scores across time among Mini Hyrax wearers were similar to those of the Hyrax wearers. In both groups, the functional limitation scores and the physical discomfort scores were significantly higher in T1 than in T0, indicating a worsening of these two domains within the 14 days after the placement of expanders (*p* < 0.016). In both groups, the handicap scores were significantly higher in T0 than in T2, indicating an improvement of this domain 6 months after the placement of expanders (*p* < 0.016). In both groups, the social disability scores and the total scores were significantly higher in T1 than in T2, indicating an improvement 6 months after the activation of the expander (*p* < 0.016). The inter-group comparisons demonstrated no difference between groups with respect to the OHIP-14 scores at T1 and OHIP-14 scores at T2, controlling for the scores at T0 (*p* > 0.05).

**Table 3:** Comparison of OHIP scores at T0 between Mini Hyrax wearers and Hyrax wearers

	Mini Hyrax T0	Hyrax T0	*p* value^a^
	Mean (SD)	Mean (SD)	
FL	0.73 (1.22)	0.73 (1.10)	1.000
FD	2.47 (1.80)	1.80 (1.14)	0.240
PD	3.87 (2.41)	2.53 (2.16)	0.123
FD	0.73 (1.16)	0.47 (0.74)	0.461
PD	3.13 (2.29)	1.27 (1.38)	0.012
SD	1.60 (1.84)	0.60 (1.12)	0.086
HC	0.80 (1.14)	0.40 (0.73)	0.265
SCO	13.33 (8.04)	7.80 (6.80)	0.051

T0: pretreatment

^a^Student *t* test (independent samples)

*SD* standard deviation, *FL* functional limitation, *FD* physical discomfort, *PD* psychological discomfort, *FD* physical disability, *PD* psychological disability, *SD* social disability, *HC* handicaps, *SCO* total score

**Table 4:** Intragroup and intergroup comparison of the 7 dimensions and the total score of the OHIP-14

Dimensions OHIP 14	T0 Mean (SD)	T1 Mean (SD)	T2 Mean (SD)	Statistical difference T0-T1-T2	T0 Mean (SD)	T1 Mean (SD)	T2 Mean (SD)	Statistical difference T0–T1–T2	Statistical difference Coef. (95% CI) ***p*** value	Statistical difference Coef. (95% CI) ***p*** value
Mini Hyrax					Hyrax				Mini Hyrax x Hyrax (T1 × T0)	Mini Hyrax x Hyrax (T2 × T0)
FL	0.73 (1.22)^a^	2.53 (2.06)^b^	1.93 (1.28)^ab^	0.031^*^	0.73 (1.10)^a^	2.60 (1.18)^b^	2.13 (1.88)^ab^	0.010^*^	0.06 (− 1.21–1.34) 0.916^**^	0.20 (− 1.02–1.42) 0.740^***^
FD	2.47 (1.80)^a^	3.93 (2.15)^b^	2.87 (2.03)^ab^	0.048^*^	1.80 (1.14)^a^	3.87 (1.76)^b^	2.40 (1.50)^a^	0.001^*^	0.13 (− 1.36–1.63) 0.859^**^	− 0.50 (− 1.90–0.88) 0.463^***^
PD	3.87 (2.41)^a^	2.40 (2.61)^a^	1.60 (2.26)^a^	0.088^*^	2.53 (2.16)^a^	2.47 (2.13)^a^	2.00 (1.55)^a^	0.646^*^	0.26 (− 1.61–2.14) 0.774^**^	0.32 (− 1.22–1.68) 0.673^***^
FD	0.73 (1.16)^a^	0.87 (1.06)^a^	0.47 (0.83)^a^	0.391^*^	0.47 (0.74)^a^	1.93 (2.05)^a^	1.00 (1.36)^a^	0.065^*^	1.07 (− 0.18–2.33) 0.091^**^	0.59 (− 0.26–1.44) 0.167^***^
PD	3.13 (2.29)^a^	2.20 (1.74)^a^	1.53 (1.76)^a^	0.196^*^	1.27 (1.38)^a^	1.47 (1.76)^a^	0.67 (0.72)^a^	0.051^*^	− 0.94 (− 2.43–0.54) 0.206^**^	− 1.15 (− 2.28–0.03) 0.052^***^
SD	1.60 (1.84)^ab^	1.20 (1.74)^a^	0.53 (0.99)^b^	0.038^*^	0.60 (1.12)^ab^	0.67 (1.17)^a^	0.13 (0.35)^b^	0.009^*^	− 0.31 (− 1.48–0.85) 0.583^**^	− 0.32 (− 0.91–0.26) 0.272^***^
HC	0.80 (1.14)^a^	0.40 (0.82)^ab^	0.00 (0.00)^b^	0.030^*^	0.40 (0.73)^a^	0.40 (0.82)^ab^	0.07 (0.25)^b^	0.049^*^	0.02 (− 0.61–0.67) 0.930^**^	0.06 (− 0.08–0.20) 0.390^***^
SCO	13.33 (8.04)^ab^	13.53 (7.12)^a^	8.93 (5.67)^b^	0.026^*^	7.80 (6.80)^ab^	13.40 (8.49)^a^	8.33 (5.86)^b^	0.040^*^	− 0.08 (− 6.49–6.32) 0.978^**^	− 0.70 (− 5.41–4.01) 0.762^***^

*T0* pretreatment, *T1* 14th day of the appliance activation, *T2* after 6 months retention period, *SD* standard deviation, *FL* functional limitation, *FD* physical discomfort, *PD* psychological discomfort, *FD* physical disability, *PD* psychological disability, *SD* social disability, *HC* handicaps, *SCO* total score

^*^ANOVA test for repeated measures, Level of significance *p* < 0.05

For comparison of pairs T0 X T1, T0 X T2, T1 X T2 in Mini-Hyrax group and Hyrax group, paired *t* test was employed. Different letters indicate significant difference. Bonferroni correction. Level of significance *p* < 0.016

*Coef* coefficient

^**^Regression analysis assessing differences between Mini-Hyrax and Hyrax groups of the OHIP-14 scores at T1, controlling for the OHIP-14 scores at T0. Level of significance *p* < 0.05

***Regression analysis assessing differences between Mini-Hyrax and Hyrax groups of the OHIP-14 scores at T2, controlling for the OHIP-14 scores at T0. Level of significance *p* < 0.05

The highest scores related to the perception of pain during the activation phase of the devices were found in 24 h and from this point forward, there was a reduction in scores up to 7 days for both expander wearers. The pain reduction in the Mini Hyrax group was gradual over the four times, while in the Hyrax group, there was a significant reduction between 48 h and 72 h. Considering the inter-group comparison for each of the four observation times, no statistical difference was observed (Table 5).

**Table 5:** Intragroup and intergroup comparison of the pain perception using the visual analogical scale (VAS)

Observational times	Groups						Effect size (95% CI)	***p*** value
	Mini Hyrax			Hyrax				
	Median	Min–Max	Mean	Median	Min–Max	Mean		
24 h	45.30	0.00–96.80	37.35 A	35.57	0.00–91.84	38.96 A	0.05 (− 4.00–4.10)	0.847
48 h	27.19	0.00–91.37	35.46 A	27.72	0.00–83.44	31.06 A	0.16 (− 0.54–0.86)	0.653
72 h	15.77	0.00–93.56	25.63 AB	9.44	0.00–75.78	22.37 B	0.12 (− 0.58–0.82)	0.967
7 days	8.22	0.00–71.59	15.32 B	4.85	0.00–64.77	16.21 B	0.04 (− 3.23–3.31)	0.844

Intergroup comparison (Mini Hyrax versus Hyrax): Mann-Whitney test. Level of significance *p* < 0.05

Intragroup comparison (between pairs of time): Wilcoxon test. Level of significance *p* < 0.05.

Same letters: not significative statistical difference. Different letters: significant statistical

*CI* confidence interval

### Harms

Gingival retraction, white spot lesion, and severe pain were not identified in the participants. The side effect observed was inflammation of the palatal marginal gingiva of the first premolars in four Hyrax wearers and three Mini Hyrax wearers, due to the displacement of the anterior portion of the devices against the gingiva. This change was completely reversed after removing the expanders. In 1 individual among the 15 wearing Hyrax and in 1 among the 15 wearing Mini Hyrax, the predetermined transverse overcorrection was not obtained due to the expansion limit of the jackscrews. These two subjects were included in the sample and shortly after the observation period, a second expansion was carried out.

## Discussion

According to systematic reviews published in recent years, scientific evidence on RPE is basically limited to the assessment of the effects of orthodontic expanders on hard and soft tissues of the stomatognathic system [22–25]. Researchers have not fully explored the impact of these interventions on the quality of life of individuals [11, 14]. Orthodontic interventions should be not only efficacious in treating malocclusion, but they should also cause minimal pain and minimal impact on patients' quality of life [26].

The present study was not restricted to the evaluation of the dental effects of Hyrax and Mini Hyrax. The assessments of the impact on the quality of life and the pain perception of patients contributed to reduce the gap of knowledge on orthodontic devices used in RPE. In addition, this is the first randomized controlled clinical trial, in which the Mini Hyrax has been evaluated.

The wearing of both expanders increased the transverse distances of premolars and molars, varying from 5.93 to 6.55 mm, similar to what has been reported elsewhere [22–25]. The tooth rotation varied from − 1.05^o^ to 1.77^o^, without a specific direction for each type of expander and without a statistically significant difference between them, as reported in another study [27], in which tooth rotation was minimal and did not provoke any relevant clinical drawbacks. The effect on canines was not in the scope of this investigation because maxillary constriction is very much associated with lack of space in the maxilla arch and impaction of upper canines is a common finding [28].

There was an increase in the buccal inclination of premolars, from 9.25^o^ to 12.17^o^, and of molars, from 1.53^o^ to 2.79^o^. Herein, the magnitude of the increase in the buccal inclination of premolars was greater than the findings of the literature [4, 24, 29]. The difference between premolars and molars is probably justified by the greater proximity of the jackscrew to the molars' center of resistance. The premise that the molars were banded and the premolars were bonded may also have influenced the results [4]. The only statistically significant difference between individuals wearing Hyrax and Mini Hyrax was for the upper left second premolar. However, the mean difference was only 2.70^o^, with no relevant clinical significance.

Several studies have used the palatal rugaes as reference structures in model superimpositions for assessing changes in tooth position resulting from growth and aging as well as orthodontic treatment [18, 30–34]. The medial point of the third rugae and the medial of the second rugae, in this order, have been determined as the most stable points [30, 31, 33, 35]. However, there are no definitely stable points in the oral cavity [33, 36, 37] and the accuracy of point-based model superimposition is lower than area-based landmark superimposition [18, 33].

The 3D Slicer software allows an operator to select an area by means of the determination of reference points and the size of the regions of interest (ROI), according to the reliability of each region of the palate for the type of tooth movement evaluated in the model superimposition. Thus, the medial end of the second and third rugaes was selected. No point was marked on the first rugae because changes in the position of the incisors during RPE, such as incisor extrusion and palatal inclination [27] cause changes in this rugae [35, 38]. The two most distal points located on the median palatal raphe were included to increase the control of the “pitch” [18, 34]. The ROI size of 15 was defined for all points, determining the area of superimposition in the middle of the palate to avoid lateral displacement (“roll” control) of the pre- and posttreatment models by the “best-fit.” This method has been adopted because the lateral inclination of the palatal vault changes with expansion [30, 39].

Our trial demonstrated that the impact on quality of life across time among Mini Hyrax wearers was very much alike to that of the Hyrax wearers. The worsening of function and discomfort 14 days after the bonding of the expander may be explained by the placement of the orthodontic device itself and the activation of screw and forces applied for the expansion. The improvement in handicap, social disability and the overall quality of life 6 months after treatment onset may be due to the recognition of the adolescent that he/she is on the way towards malocclusion treatment and the wearing of an orthodontic device is perceived as a normal circumstance over the course of treatment. This information is useful to clinicians during the counseling of adolescents wearing rapid maxillary expanders and their parents/guardians [40].

The absence of differences in the impact on quality of life between the wearers of both orthodontic expanders (inter-group comparison) may be related to the vertical position of the expander jackscrew. The small size of Mini Hyrax, initially considered an advantage, may also represent a limitation, if the jackscrew is placed too far from the palatal vault, since there is less area of contact between the device and the tongue.

The vertical position of the jackscrew can have influence on bone expansion and on the type of movement of the anchored teeth [41, 42]. From an orthopedic point of view, the ideal position of the jackscrew should be slightly higher than the center of resistance of the molars, mitigating buccal dental inclination [41]. However, in cases of more severe maxillary atresia, this ideal jackscrew position could be a shortcoming when the jackscrew is welded to the expander device. In our study, the vertical position of the jackscrew was close to the resistance center of the first molars.

The scores of pain perception of the wearers of Mini Hyrax reduced from 24 h to 7 days during the activation of the expander, with no significant statistical differences between consecutive observation intervals. Indeed, the reduction in pain perception between the observation times was subtle and only between 24 h and 7 days, a significant difference was observed. On the other hand, pain perception among Hyrax wearers reduced significantly between 48 h and 72 h during the activation of the expander. This information may be helpful for the clinician during the counseling of patients wearing Mini Hyrax or Hyrax regarding pain and discomfort.

### Limitations

In this study, assessment of bone maturation of participants was not carried out. It can be considered that this confounding factor might have been evenly distributed across the two groups due to the randomization; however, as long as this parameter was not measured, its possible influence on the outcomes cannot be ruled out with certainty.

Another possible limitation is the use of the palate as a reference for the digital model superimposition, since the lowering of the palate during RPE has already been reported [30, 39]. The use of computed tomography would allow an operator to perform superimposition of structures outside the oral cavity that do not change with RPE. Assessments of the cranial base, for instance, provide evaluations with greater precision. However, ethical issues preclude this type of analysis.

Finally, the number of participants in our study was determined by means of a sample size calculation and the number of losses was minimal. However, we do recognize that the present study may be underpowered to identify large, moderate, or small effects regarding the outcomes assessed herein.

## Conclusions

The null hypothesis was accepted:

1. There were no significant differences regarding dental effects during RPE between adolescents Mini Hyrax wearers and Hyrax wearers.
2. There was no significant difference regarding the impact of RPE on quality of life between adolescents wearing Mini Hyrax and those wearing Hyrax.
3. There was no significant difference regarding pain perception during RPE between adolescents wearing Mini Hyrax and those wearing Hyrax.

## Data Availability

The datasets generated and/or analyzed during the current study are available from the corresponding author on reasonable request.

## References

[1] Betts NJ, Vanarsdall RL, Barber HD, Higgins-Barber K, Fonseca RJ (1995). Diagnosis and treatment of transverse maxillary deficiency. Int J Adult Orthodon Orthognath Surg..

[2] Phillips C, Medland WH, Fields HW, Proffit WR, White RP (1992). Stability of surgical maxillary expansion. Int J Adult Orthodon Orthognath Surg..

[3] Allen D, Rebellato J, Sheats R, Ceron AM (2003). Skeletal and dental contributions to posterior crossbites. Angle Orthod..

[4] Garib DG, Henriques JFC, Janson G, Freitas MR, Coelho RA (2005). Rapid maxillary expansion - tooth tissue-borne versus tooth-borne expanders: a computed tomography evaluation of dentoskeletal effects. Angle Orthod..

[5] Suri L, Taneja P (2003). Surgically assisted rapid palatal expansion: a literature review. Am J Orthod and Dentofacial Orthop..

[6] Weissheimer A, De Menezes LM, Mezomo M, Dias DM, De Lima EM, Rizatto SM (2011). Immediate effects of rapid maxillary expansion with Haas-type and hyrax-type expanders: a randomized clinical trial. Am J Orthod and Dentofacial Orthop..

[7] Biederman W (1973). Rapid correction of class III malocclusion by midpalatal expansion. Am J Orthod..

[8] Stevens K, Bressman T, Gong S, Tompson BD (2011). Impact of a rapid palatal expander on speech articulation. Am J Orthod Dentofacial Orthop..

[9] Gecgelen M, Aksoy A, Kirdemir P, Doguc DK, Cesur G, Koskan O, Ozorak O (2012). Evaluation of stress and pain during rapid maxillary expansion treatments. J Oral Rehabil..

[10] Oliveira DD, Bartolomeo FUC, Cardinal L, Figueiredo DSF, Palomo JM, Andrade I (2014). An alternative clinical approach to achieve greater anterior than posterior maxillary expansion on cleft lip and palate patients. J Craniofac Surg..

[11] Feldmann I, Bazargani F (2017). Pain and discomfort during the first week of rapid maxillary expansion (RME) using two different RME appliances: a randomized controlled trial. Angle Orthod..

[12] Lamparski DG, Rinchuse DJ, Close JM, Sciote JJ (2003). Comparison of skeletal and dental changes between 2-point and 4-point rapid palatal expanders. Am J Orthod Dentofacial Orthop..

[13] Bratu DC, Bratu EA, Popa G, Luca M, Balan R, Ogodescu A (2012). Skeletal and dentoalveolar changes in the maxillary bone morphology using two-arm maxillary expander. Rom J Morphol Embryol..

[14] Biondi E, Bandini A, Lombardo L, Orlandi S, Siciliani G, Manfredi C (2017). Phonetic analysis during treatment with rapid maxillary. Orthod Craniofac Res..

[15] Moher D, Hopewell S, Schulz KF, Montori V, Gøtzsche PC, Devereaux PJ, Elbourne D, Egger M, Altman DG, CONSORT (2012). CONSORT 2010 explanation and elaboration: updated guidelines for reporting parallel group randomised trials. Int J Surg..

[16] De Oliveira BH, Nadanovsky P (2005). Psychometric properties of the Brazilian version of the Oral Health Impact Profile–short form. Community Dent Oral Epidemiol..

[17] Hjermstad MJ, Fayers PM, Haugen DF, Caraceni A, Hanks GW, Loge JH, Faisinger R, Aass N, Kaasa S (2011). Studies comparing numerical rating scales, verbal rating scales, and visual analogue scales for assessment of pain intensity in adults: a systematic literature review. J Pain Symptome Manage..

[18] Anacleto MA, Souki BQ (2019). Superimposition of 3D maxillary digital models using open-source software. Dental Press J Orthod..

[19] Saghaeri M (2004). Random allocation software for parallel group randomized trials. BMC Med Res Methodol..

[20] Dahlberg G (1940). Statistical methods for medical and biological students.

[21] Cohen J (1988). Statistical Power Analysis for the Behavioural Sciences.

[22] Bastos RTDRM, Blagitz MN, Aragón MLSC, Maia LC, Normando D (2019). Periodontal side effects of rapid and slow maxillary expansion: a systematic review. Angle Orthod..

[23] Cannavale R, Chiodini P, Perillo L, Piancino MG (2018). Rapid palatal expansion (RPE): meta-analysis of long-term effects. Orthod Craniofac Res..

[24] Algharbi M, Bazargani F, Dimberg L (2018). Do different maxillary expansion appliances influence the outcome of the treatment?. Eur J Orthod..

[25] Bucci R, D'Antó V, Rongo R, Valleta R, Martina R, Michelotti A (2016). Dental and skeletal effects of palatal expansion techniques: a systematic review of the current evidence from systematic reviews and meta-analyses. J Oral Rehabil..

[26] Schuttinga JA, Dimsadale JE, Baum A (1995). Quality of life from a federal regulatory perspective. Quality of life in behavioral medicine research.

[27] Canan S, Senisik E (2017). Comparison of the treatment effects of different rapid maxillary expansion devices on the maxilla an the mandible. Part 1: Evaluation of dentoalveolar changes. Am J Orthod Dentofacial Orthop..

[28] Cruz RM (2019). Orthodontic traction of impacted canines: concepts and clinical application. Dental Press J Orthod..

[29] Alves ACMA, Janson G, McNamara JA, Lauris JRP, Garib DG (2020). Maxillary expander with differential opening vs Hyrax expander: a randomized clinical trial. Am J Orthod Dentofacial Orthop..

[30] Choi JI, Cha BK, Jost-Brinkmann PG, Choi DS, Jang IS (2012). Validity of palatal superimposition of 3-dimensional digital models in cases treated with rapid maxillary expansion and maxillary protraction headgear. Korean J Orthod..

[31] Chen G, Chen S, Zhang XY, Jiang RP, Liu Y, Shi FH, Xu TM (2011). Stable region for maxillary dental cast superimposition in adults, studied with the aid of stable miniscrews. Orthod Craniofac Res..

[32] Kim HK, Moon S, Lee SJ, Park YS (2012). Three-dimensional biometric study of palatine rugae in children with a mixed-model analysis: a 9-year longitudinal study. Am J Orthod Dentofacial Orthop..

[33] Abdi AH, Nouri M (2017). Registration of serial maxillary models via the weighted rugae superimposition method. Orthod Craniofac Res..

[34] Garib D, Miranda F, Yatabe MS, Lauris JRP, Massaro C, McNamara JA, Kim-Berman H, Janson G, Behrents RG, Cevidanes LHS, Ruellas ACO (2019). Superimposition of maxillary digital models using the palatal rugae: does ageing affect the reliability?. Orthod Craniofal Res..

[35] Jang I, Tanaka M, Koga Y, Iijima S, Yozgatian J, Bk C, Yoshida N (2009). A novel method for the assessment of three-dimensional tooth movement during orthodontic treatment. Angle Orthod.

[36] Damstra J, Mistry D, Cruz C, Ren Y (2009). Antero-posterior and transverse changes in the positions of palatal rugae after rapid maxillary expansion. Eur J Orthod..

[37] Saadeh M, Macari A, Haddad R, Ghafari J (2017). Instability of palatal rugae following rapid maxillary expansion. Eur J Orthod..

[38] Christou P, Kiliaridis S (2008). Vertical growth-related changes in the positions of palatal rugae and maxillary incisors. Am J Orthod Dentofacial Orthop..

[39] Jafari A, Shetty KS, Kumar MK (2003). Study of stress distribution and displacement of various craniofacial structures following application of transverse orthopaedic forces-a three-dimensional FEM study. Angle Orthod..

[40] Alghamdi MA, Farsi NJ, Hassan AH (2017). Comparison of oral health-related quality of life of patients treated by palatal expanders with patients treated by fixed orthodontic appliances. Patient Prefer Adherence..

[41] Araugio EMS, Landre J, Silva ALA, Pacheco W, Pithon MM, Oliveira DD (2013). Influence of the expansion screw height on the dental effects of the hyrax expander: a study with finite elements. Am J Orthod Dentofacial Orthop..

[42] Fernandes LC, Vitral RWF, Noritomi PY, Schmitberger CA, Campos MJS (2019). Influence of the hyrax expander screw position on stress distribution in the maxilla: a study with finite elements. Am J Orthod Dentofacial Orthop..

